# Assessing Risk Classification in Medication-Induced Diabetes during Induction Therapy in Pediatric Acute Lymphoblastic Leukemia

**DOI:** 10.1155/2023/1057639

**Published:** 2023-12-16

**Authors:** Katie Ross, Ketan Kulkarni, Tamara MacDonald, Teresa Pinto

**Affiliations:** ^1^Faculty of Medicine, Dalhousie University, Halifax B3H 4R2, Canada; ^2^Division of Hematology/Oncology, Department of Pediatrics, IWK Health, Halifax B3K 6R8, Canada; ^3^Department of Pharmacy, IWK Health, Halifax B3K 6R8, Canada; ^4^Faculty of Health Professions, Dalhousie University, Halifax B3H 4R2, Canada; ^5^Division of Endocrinology, Department of Pediatrics, IWK Health, Halifax B3K 6R8, Canada

## Abstract

Medication-induced diabetes (MID) is common during induction therapy for pediatric acute lymphoblastic leukemia (ALL) and has potentially significant negative consequences. Reported risk factors for MID are variable with limited data comparing patients treated with standard-risk (SR) vs. high-risk (HR) regimens. This study aims to evaluate the incidence and risk factors for MID during induction in patients with ALL from the Maritimes over a 20-year period. We performed a retrospective single-center study of 262 patients (142 males, 120 females) diagnosed with ALL at IWK Health in Halifax, Nova Scotia, Canada, from 2000 to 2019. Older age, higher body mass index, greater central nervous system status, Trisomy 21, and prednisone steroid type were risk factors associated with MID in our cohort. HR patients developed significantly more complications than SR patients including MID and infection. Screening for MID should be routine during ALL induction treatment, particularly in those with HR disease.

## 1. Introduction

Acute lymphoblastic leukemia (ALL) is the most common type of cancer in children [1]. At diagnosis, patients are categorized as standard-risk (SR) or high-risk (HR) based on various prognostic factors outlined by the National Cancer Institute (NCI) [1, 2]. During induction therapy, SR patients receive asparaginase, vincristine, and a steroid (three-drug regimen), whereas HR patients receive an additional anthracycline medication (four-drug regimen) [2, 3]. Despite being the accepted treatment, morbidity from pediatric ALL therapy remains significant [4, 5].

One common side effect during induction treatment is medication-induced diabetes (MID). MID is defined as the development of a hyperglycemic state that meets the definition of diabetes after the ingestion of a medication known to cause hyperglycemia [6]. Though most ALL patients will have a transient form of MID, many can still experience significant negative sequelae, such as increased infectious complications and poorer survival [7–9].

Reported incidence rates of MID in pediatric ALL patients range from 5.6% to 20.4% [7, 10–12]. Patient risk factors that may be associated with MID include older age at diagnosis, higher body mass index (BMI), female gender, central nervous system (CNS) disease, and Trisomy 21 [7, 9, 10]. Though studies of pediatric ALL patients have identified some potential risk factors for MID, very few have assessed the incidence of MID in patients receiving SR (three drugs) versus HR (four drugs) induction chemotherapy.

The purpose of the current study is to evaluate the incidence and risk factors for MID during induction treatment in pediatric patients with ALL in the Canadian Maritime Provinces (including Nova Scotia, New Brunswick, and Prince Edward Island) over a 20-year period.

## 2. Materials and Methods

### 2.1. Study Population

Patients for this retrospective chart review were identified from the Pediatric Oncology Research Database at IWK Health in Halifax, Nova Scotia. Subjects were less than 19 years of age and diagnosed with ALL (B or T cells) between February 2000 and December 2019. All subjects received their induction chemotherapy at the IWK and were managed primarily at the IWK in a shared care model with regional provincial hospitals.

Patients were categorized at diagnosis using the NCI definitions for SR and HR ALL, with SR patients being age 1–9.99 years with a white blood count (WBC) less than 50,000/*µ*L and HR patients being age 10 years or older and/or with a WBC 50,000 *µ*L or greater [4]. Infants younger than 1 year were a special subgroup of patients and considered HR [4]. Patients with preexisting diabetes were excluded.

Patients were treated according to protocols from the Children's Cancer Group, Children's Oncology Group, or Pediatric Oncology Group. The typical treatment regimen that SR and HR patients received is listed in Tables 1 and 2. Although several different protocols were followed, all used the same SR and HR medications with similar doses during the induction phase. Protocols varied during the consolidation and maintenance phases, but they were not included in this study. All patients received pegylated (PEG) asparaginase during induction therapy. Lastly, steroid dosing was body-surface area based and did not have an upper cut-off limit, so steroid dose increased proportionally with weight.

To screen for MID, patients' urine glucose was measured twice weekly. If found to be elevated, blood glucose was drawn. Patients were only diagnosed with MID if they met the definition of diabetes outlined by the International Society of Pediatric and Adolescent Diabetes. This includes a random serum glucose level ≥11.1 mmol/L or a fasting serum glucose level ≥7.0 mmol/L with symptoms or two random serum glucose levels ≥11.1 mmol/L or two fasting serum glucose levels ≥7.0 mmol/L obtained on separate days without symptoms. This definition is consistent with previous studies of MID in pediatric ALL patients [10, 13, 14].

Infective episodes were defined by positive laboratory findings and recorded as either bacterial, viral, fungal, or parasitic. Only infective episodes that occurred during induction therapy were included. CNS status was classified into three categories: CNS1, CNS2, and CNS3. This was based on the presence and level of leukemic cells in cerebral spinal fluid and absolute cell counts [15].

For patients older than 2 years of age, BMI was calculated using weight in kilograms/height in meters^2^ [2]. WHO Growth Charts for Canada, age 2–19, were used to calculate BMI percentiles. The Merck Manuals calculator was then used to calculate *z*-scores.

For patients under 2 years of age, WHO Growth Charts for Canada—Infant Weight for Length were used to calculate growth percentiles. The Merck Manuals calculator was then used to calculate *z*-scores.

This study was approved by the IWK ethics board.

### 2.2. Study Procedure

Demographic data were extracted from patients' electronic health records. Information recorded included age at diagnosis, gender, height (cm), and weight (kg) at diagnosis, serum glucose levels, type of steroid, type of asparaginase, infection during treatment, type of infection, relapsed disease, and death. Patients with MID were flagged for further chart review.

### 2.3. Statistical Analyses

Descriptive statistics were calculated for demographic and clinical variables. Comparisons were made between SR and HR patients, with and without MID, during ALL induction treatment. Student *t*-test and ANOVA were used to compare the means of continuous variables, and *χ*^2^-test was used for categorical variables. For all comparisons, *p* < 0.05 was considered statistically significant.

## 3. Results

The cohort included 262 eligible patients.  Table 3 summarizes the demographic and clinical characteristics of the study population with statistically significant values bolded. One hundred forty-two (54.2%) patients were male, median age at diagnosis was 6.55 years (range 0.08–18.75), and mean BMI *z*-score was 0.702 (range −3.97 to 2.92). Majority of patients had B-cell subtype ALL (92.3%).

Twenty-two patients (8.4%) developed MID during induction therapy. Of those who developed MID, 15 (68.2%) received treatment with insulin, three (13.6%) did not require treatment, and information was unavailable for four patients. Patients who developed MID were significantly older (10.3 vs. 6.2 years, *p* < 0.001), had higher BMI *z*-scores (1.2 vs. 0.3, *p*=0.003), and had higher rates of Trisomy 21 (9.5% vs. 1.3%, *p*=0.012) than those who did not. Patients with MID had significantly higher CNS status (*p* < 0.001) but did not have increased rates of infection, relapsed disease, or death (Table 3). Majority of patients with MID received prednisone (63.6%), whereas most patients without MID received dexamethasone (76.2%).

Demographic and clinical characteristics by risk classification are also detailed in Table 3. As expected from NCI criteria, all SR patients were less than 10 years of age and 54% of HR patients were greater than 10 years of age. As a result, the average age of HR patients was significantly older than SR patients (10.25 vs. 5.10, *p* < 0.001). HR patients also had significantly higher BMI *z*-scores (0.58 vs. 0.15, *p*=0.010) and significantly more complications than SR patients including MID (13.1% vs. 4.3%, *p*=0.010), higher CNS status (*p*=0.007), infection rate (68.3% vs. 45.7%, *p* < 0.001), number of infections (2.38 vs. 1.80, *p* < 0.001), relapsed disease (10.7% vs. 4.3%, *p*=0.047), and death (11.5% vs. 1.4%, *p* < 0.001). Most SR patients received dexamethasone as their steroid (98.5%), whereas majority of HR patients received prednisone (53.0%). Two SR and one HR patient developed pancreatitis during induction therapy.

Table 4 compares patients by risk classification and MID diagnosis with statistically significant values bolded. There was no significant difference in age, sex, BMI, or CNS status in SR patients with MID vs. without. HR patients who developed MID were significantly older (12.09 vs. 8.42, *p*=0.014), had higher BMI *z*-scores (1.26 vs. 0.48, *p*=0.003), and had higher CNS status (*p*=0.010) compared to HR patients without MID. Although not significant, HR patients with MID developed hyperglycemia sooner after induction therapy initiation compared to SR patients with MID (6.56 days vs. 9.50 days, *p*=0.093). Thirty-five percent of HR T-cell ALL patients were >10 years of age compared to 48% of HR B-cell ALL patients.

Lastly, Table 5 compares HR patients treated with dexamethasone vs. prednisone with statistically significant values bolded. Overall, HR patients treated with prednisone were significantly older than those treated with dexamethasone (13.33 vs. 3.49, *p* < 0.001) and developed significantly more MID (21.5% vs. 3.5%, *p*=0.003). When specifically analyzing HR patients over 10 years of age who received prednisone, those who developed MID, had significantly higher BMI *z*-scores (1.32 vs. 0.39, *p*=0.0017) than those who did not.

## 4. Discussion

MID is a common side effect during pediatric ALL treatment. In our cohort, 8.4% of patients developed MID during the induction phase of treatment, and 83% of these patients required treatment with insulin.

The occurrence of MID during therapy has been mainly attributed to acute stress, critical illness, and the direct effect of chemotherapeutic agents [16, 17]. L-asparaginase has been shown to cause subclinical impairment of insulin secretion, and glucocorticoids have been associated with multiple mechanisms including *β* cell damage, decreased insulin synthesis, increased insulin resistance, and increased gluconeogenesis [17, 18]. When used alone or particularly in combination, these agents can cause hyperglycemia in the diabetes range [19]. Of note, MID is not limited to patients with ALL. Steroids used for treatment in other acute and chronic conditions such as nephrotic syndrome and collagen disease report rates of steroid-induced hyperglycemia that range from 2% to 26% [20, 21].

When comparing risk groups, 4% of SR patients developed MID versus 13% of HR patients. This is similar to West et al.'s [22] study who reported that 4% of SR patients developed hyperglycemia versus 18% of HR patients in their cohort. However, they graded hyperglycemia using the Common Terminology Criteria for Adverse Events (CTCAE). They used a grade 2 or higher as their cut-off, indicating patients had received insulin or other medications to treat the hyperglycemia. Although the CTCAE is able to indicate the severity of adverse events commonly encountered in oncology, our method of using blood glucose levels to diagnosis MID is the most common method used in published literature on hyperglycemia in pediatric ALL. This allows us to make comparisons to previous literature using the internationally accepted definition of diabetes.

Tsai et al. [23] also studied risk factors for hyperglycemia during chemotherapy for pediatric ALL in Taiwanese children. They found that 4% of SR patients and 24% of HR patients developed MID [23]. However, they recorded hyperglycemia throughout the full length of treatment and not exclusively during induction therapy. Lastly, Sonabend et al. [8] reported the rates of hyperglycemia by risk classification, but included postprandial hyperglycemia readings and reported both mild and overt hyperglycemia. Mild hyperglycemia included patients who had two or more blood glucose concentrations between 7.8 and 11.1 mmol/L, while overt hyperglycemia included those with one or more blood glucose concentrations greater than 11.1 mmol/L [9]. Their rates were higher with 25% and 18% of SR patients having mild and overt hyperglycemia and 24% and 56% of HR patients having mild and overt hyperglycemia, respectively [9].

The majority of patients who developed MID in our cohort received prednisone as their steroid. When separated by risk classification, HR patients treated with prednisone developed the highest rate of MID, with more than one in five patients developing high blood sugars. Patients who developed MID were also found to be significantly older and have higher BMI *z*-scores. McCormick et al. [24] analyzed more than 6,000 pediatric ALL patients in the United States. Similarly, older children were typically treated with prednisone in their treatment protocols. They identified an association between hyperglycemia and prednisone use on univariate analysis, but this relationship was not significant on multivariate analysis [24]. The loss of an association between prednisone use and hyperglycemia may represent confounding by the influence of age and HR status [24]. Therefore, it is difficult to conclude if it is the higher disease burden responsible for hyperglycemia in this group or age and steroid type used or a combination of any of these.

Regarding long-term outcomes of patients, only one HR patient required long-term insulin replacement for persistent diabetes. This patient was investigated for type 1 diabetes which found GAD antibodies to be negative.

Asparaginases are a critical component of pediatric ALL therapy. This chemotherapeutic agent works by depleting serum asparagine and selectively killing leukemic cells that require this critical amino acid for protein synthesis. There are two native forms: one derived from *Escherichia coli* and the other from *Erwinia chrysanthemi*. Erwinia asparaginase has a half-life of approximately 0.6 days, and *E. coli* has a half-life of approximately 1.2 days [25]. Monomethoxypolyethylene glycol (PEG) is also frequently covalently attached to *E. coli* asparaginase, extending its half-life to 5.7 days [25]. Because of the need for less frequent dosing, pegylated asparaginase is the first line choice and all of our patients received this form during induction therapy. However, up to 30% of patients develop a hypersensitivity reaction and need to be switched to Erwinia [26]. The mean time to development of MID after receiving asparaginase was 2.56 days, which was comparable to Pollock et al. [27] who found that mean glucose levels peaked 3 days following administration of PEG-asparaginase during the induction phase of treatment in their cohort of children with ALL.

CNS status is determined at diagnosis and classified as CNS 1, 2, or 3. Those with CNS 2 or 3 receive more intense treatment with increased intrathecal doses of cytarabine and methotrexate during induction therapy. In our population, greater CNS disease was associated with higher rates of MID. Neither IT cytarabine nor methotrexate is not known to cause hyperglycemia, and in fact, use of methotrexate may have some protective effects against insulin resistance [28]. Given the small sample size, however, it cannot be concluded that CNS disease increases risk of MID independently.

In our cohort, patients who developed MID were also more likely to have Trisomy 21. Patients with Trisomy 21 are also known to have higher rates of diabetes in the noncancer population, and these findings are consistent with the current literature [7, 9, 22]. Our patients with MID did not have increased rates of infection, relapsed disease, or death. We also did not find a relationship between sex and MID. These results oppose some published data on MID in pediatric ALL patients [7–9, 22, 29, 30].

Lastly, it is important to discuss the testing protocol used in our population. Typically, patients' urine glucose was measured twice weekly and if elevated, blood glucose was drawn. However, these glucose checks were not part of a standardized protocol. Handattu et al. [17] proposed an approach to diagnosis and management of MID in pediatric ALL patients. They highlighted the importance of considering multiple factors when screening for hyperglycemia including the potency of steroids used, the dose, duration and peak time to action, dose scheduling, individual risk factors, and concurrent use of other medications [17]. For example, prednisone is an intermediate-acting glucocorticoid. When given as a single morning dose, it is likely to cause postlunch and nighttime hyperglycemia corresponding to its peak (4–6 hr) and duration of action (12–16 hr). Therefore, glucose measurement should be postlunch and/or predinner [17]. On the other hand, dexamethasone is a long-acting steroid. This will result in persistent hyperglycemia throughout the day with a slight decrease after fasting overnight. A fasting glucose is therefore ideal to test for hyperglycemia in these patients. For L-asparaginase-induced MID, a random blood glucose test can be used as there is no fixed pattern of hyperglycemia [17]. This testing is especially important during induction therapy as medications are given in high doses.

This study is the first to look at the entire population of patients diagnosed with ALL in the Maritime provinces over a 20-year time span. A strength of this study includes the standardized approach to treatment: one health center directed the therapy for all children and had a long-term patient follow-up to assess disease relapse and long-term survival. Although single-center studies risk not being generalizable to other populations, patients seen at this center represented patients from all over the Canadian Maritime provinces, with an estimated total population of 1.9 million, and therefore is a diverse cohort. One limitation was the format of glucose checks. These were not conducted as part of a standardized protocol and may have underestimated the prevalence of MID in our cohort. The sample size of this study is another major limitation. Because of our sample size, we were unable to do multivariate analyses to evaluate the impact of various factors independently. We also know that family history of diabetes mellitus can have an impact on occurrence of hyperglycemia. This information was not always documented in the charts for review. Finally, patients were treated over a 20-year time span. A variety of treatment protocols were used and although the same chemotherapeutic drugs were administered, the doses and schedule varied slightly. We were therefore not able to assess the effect of any one protocol on the development of MID.

## 5. Conclusions

In conclusion, this study demonstrates that hyperglycemia during induction chemotherapy is associated with HR disease, prednisone use, older age, higher BMI, higher CNS status, and Trisomy 21. As hyperglycemia can be easily identified through routine screening, protocols that consider individual patient risk factors and treatment regimens should be developed and implemented, particularly for HR leukemia patients receiving prednisone. For example, assessing risk at the beginning of treatment would be important and higher risk individuals may warrant more frequent monitoring during the induction phase. In addition, routine fasting glucose or urinalysis during the induction phase that is standardized for all patients would be necessary. Future studies are needed to evaluate the impact of standardized early identification and treatment on patient health outcomes.

## Figures and Tables

**Table 1:** Standard risk B- and T-ALL induction phase (35 days) regimens.

Chemotherapy agents	Schedule	Dose
Dexamethasone PO	Days 1–28	6 mg/m^2^
Vincristine IV	Days 1, 8, 15, 22	1.5–2 mg/m^2^
PEG asparaginase IM/IV	Day 4	2,500 IU/m^2^
Cytarabine IT	Day 1 (CNS2 requires more)	30, 50, or 70 mg^a^
Methotrexate IT	Days 8, 29 (only if CNS2)	8, 10, or 12 mg^a^

^a^Doses adjusted based on risk classification. CNS, central nervous system.

**Table 2:** High-risk B- and T-ALL induction phase (35 days) regimens.

Chemotherapy agents	Schedule	Dose
Dexamethasone PO (<10 years of age)^b^	Days 1–14	10 mg/m^2^
Prednisone PO (≥10 years of age)	Days 1–28	60 mg/m^2^
Vincristine IV	Days 1, 8, 15, 22	1.5–2 mg/m^2^
Daunorubicin IV	Days 1, 8, 15, 22	25 mg/m^2^
PEG asparaginase IM/IV	Day 4	2,500 IU/m^2^
Cytarabine IT	Day 1 (CNS2 requires more)	30, 50, or 70 mg^a^
Methotrexate IT	Days 8, 29 (also days 15, 22 for CNS3)	8, 10, or 12 mg^a^

^a^Dosing adjusted for age. ^b^As dexamethasone is approximately sixfold as potent as prednisone, both HR patients under and over 10 years of age received the equivalent of 60 mg/m^2^ of prednisone, but for different durations (14 vs. 28 days). CNS, central nervous system.

**Table 3:** Demographic and clinical characteristics of study population.

Variables	All patients	No MID	MID	*p*-Value	Standard risk	High riisk	*p*-Value
	*N* = 262	*N* = 240	*N* = 22		*N* = 140	*N* = 122	
Mean age at *dx*, years (range)	6.55 (0.08–18.75)	6.21 (0.08–18.75)	10.3 (0.83–18.5)	**<0.001**	5.10 (1.0–13.3)	10.25 (0.08–18.75)	**<0.001**
Age < 10 years, *n* (%)	206 (78.6)	196 (81.7)	10 (45.5)	**<0.001**	140 (100)	66 (54.1)	**<0.001**
Age > 10 years, *n* (%)	56 (21.4)	44 (18.3)	12 (54.5)	—	0	56 (45.9)	—
Mean BMI, *z*-score (range)	0.70 (−3.97 to 2.92)	0.26 (−3.97 to 2.92)	1.16 (0.05–2.59)	**0.003**	0.15 (−3.97 to 2.92)	0.58 (−1.78 to 2.59)	**0.01**
Sex, *n* (%)					79 (56.4)	63 (51.6)	0.44
Male	142 (54.2)	131 (54.6)	11 (50)	0.68	61 (43.6)	59 (48.4)	—
Female	120 (45.8)	109 (45.4)	11 (50)		4 (2.6)	1 (0.82)	**<0.001**
Trisomy 21, *n* (%)	5 (1.9)	3 (1.25)	2 (9.1)	**0.012**	—	—	—
Type of ALL, *n* (%)					140 (100)	102 (83.6)	**<0.001**
B cell	242 (92.3)	222 (92.5)	20 (90.9)	0.79	0	20 (16.4)	—
T cell	20 (7.6)	18 (7.5)	2 (9.1)	—	—	—	—
Steroid, *n* (%)					138 (98.6)	57 (46.7)	**<0.001**
Dexamethasone	195 (74.4)	183 (76.2)	8 (36.4)	**<0.001**	2 (1.4)	65 (53.3)	—
Prednisone	67 (25.6)	57 (23.8)	14 (63.6)	—	—	—	—
CNS status, *n* (%)							
CNS 1	219 (83.6)	205 (85.4)	14 (63.6)	**<0.001**	127 (90.7)	92 (75.4)	**0.007**
CNS 2	40 (15.3)	34 (14.2)	6 (27.3)	—	13 (9.3)	27 (22.1)	—
CNS 3	3 (1.14)	1 (0.42)	2 (9.1)	—	0	3 (2.5)	—
Developed pancreatitis	3	2	1	0.129	2	1	0.644
Patients with ≥1 infection, *n* (%)	148 (56.5)	134 (55.6)	14 (63.6)	0.47	64 (45.7)	84 (68.3)	**<0.001**
Mean number of infective episodes	2.13	2.13	2.14	0.976	1.80	2.38	**<0.001**
Relapsed disease, *n* (%)	19 (7.2)	18 (7.5)	1 (4.5)	0.612	6 (4.3)	13 (10.7)	**0.047**
Deceased, *n* (%)	16 (6.1)	15 (6.2)	1 (4.5)	0.752	2 (1.4)	14 (11.5)	**<0.001**

CNS, central nervous system. Bold signifies statistically significant values.

**Table 4:** Demographic and clinical characteristics by risk classification and MID diagnosis.

Variables	Standard risk			High risk		
	No MID	MID	*p*-Value	No MID	MID	*p*-Value
	*N* = 134	*N* = 6		*N* = 106	*N* = 16	
Mean age at *dx*, years (range)	4.67 (1.0–13.3)	5.52 (3.58–9.5)	0.37	8.42 (0.08–18.75)	12.09 (0.83–18.5)	**0.014**
Age < 10 years, *n* (%)	134 (100)	6 (100)	—	62 (58.5)	4 (25)	**0.012**
Age > 10 years, *n* (%)	0	0	—	44 (41.5)	12 (75)	**—**
Mean BMI, *z*-score (range)	0.10 (−3.97 to 2.92)	0.97 (0.3–2.18)	0.196	0.48 (−1.78 to 2.50)	1.26 (0.05–2.59)	**0.003**
Sex, *n* (%)	*N* (%)	*N* (%)		*N* (%)	*N* (%)	
Male	75 (56.0)	4 (66.7)	0.605	56 (52.8)	7 (43.7)	0.498
Female	59 (44.0)	2 (33.3)	—	50 (47.2)	9 (56.3)	—
Trisomy 21, *n* (%)	3 (2.2)	1 (16.7)	**0.038**	0	1 (6.2)	**0.010**
Type of ALL, *n* (%)						
B cell	134 (100)	6 (100)	1.0	88 (83.0)	14 (87.5)	0.652
T cell	0	0	—	18 (17.0)	2 (12.5)	—
Steroid, *n* (%)						
Dexamethasone	132 (98.5)	6 (100)	0.76	51 (48.1)	2 (12.5)	**0.007**
Prednisone	2 (1.5)	0 (0)	—	55 (51.9)	14 (87.5)	
First day of hyperglycemia	—	9.50	—	—	6.56	0.093
CNS status, *n* (%)						
CNS 1	122 (91.0)	5 (83.3)	0.52	83 (78.3)	9 (56.3)	**0.010**
CNS 2	12 (9.0)	1 (16.7)	—	22 (20.7)	5 (31.2)	—
CNS 3	0	0	—	1 (0.94)	2 (12.5)	—
Patients with ≥1 infection, *n* (%)	60 (44.8)	4 (66.7)	0.28	74 (69.2)	10 (62.5)	0.54
Mean number of infective episodes	1.80	1.75	0.47	2.39	2.30	0.61
Relapsed disease, *n* (%)	5 (3.7)	1 (26.7)	0.13	13 (12.3)	0	0.14
Deceased, *n* (%)	2 (1.5)	0	0.76	13 (12.3)	1 (6.2)	0.48

CNS, central nervous system. Bold signifies statistically significant values.

**Table 5:** High-risk patient demographics by steroid treatment.

Variables	HR treated with dexamethasone	HR treated with prednisone	*p*-Value
	*N* = 57	*N* = 65	
Developed MID, *n* (%)	2 (3.51%)	14 (21.53%)	**0.003**
Mean age at *dx*, years (range)	3.49 (0.08–8.50)	13.33 (7.42–18.75)	**0.001**
Mean BMI, *z*-score (range)	0.579 (−1.19 to 1.85)	0.57 (−1.78 to 2.59)	0.544
Sex, *n* (%)	*N* (%)	*N* (%)	—
Male	30 (52.6)	33 (50.77)	0.837
Female	27 (47.4)	32 (49.23)	—

Bold signifies statistically significant values.

## Data Availability

The data used to support the findings of this study are included within the article.
